# Thiol-based redox regulation in sexual plant reproduction: new insights and perspectives

**DOI:** 10.3389/fpls.2013.00465

**Published:** 2013-11-14

**Authors:** Jose A. Traverso, Amada Pulido, María I. Rodríguez-García, Juan D. Alché

**Affiliations:** ^1^Estación Experimental del Zaidín, Consejo Superior de Investigaciones CientíficasGranada, Spain; ^2^Departamento de Fisiología Vegetal, Universidad de GranadaGranada, Spain

**Keywords:** redox regulation, sexual plant reproduction, thioredoxin, glutaredoxin, pollen, pistil, pollen-pistil interaction, stigma

## Abstract

The success of sexual reproduction in plants involves (i) the proper formation of the plant gametophytes (pollen and embryo sac) containing the gametes, (ii) the accomplishment of specific interactions between pollen grains and the stigma, which subsequently lead to (iii) the fusion of the gametes and eventually to (iv) the seed setting. Owing to the lack of mobility, plants have developed specific regulatory mechanisms to control all developmental events underlying the sexual plant reproduction according to environmental challenges. Over the last decade, redox regulation and signaling have come into sight as crucial mechanisms able to manage critical stages during sexual plant reproduction. This regulation involves a complex redox network which includes reactive oxygen species (ROS), reactive nitrogen species (RNS), glutathione and other classic buffer molecules or antioxidant proteins, and some thiol/disulphide-containing proteins belonging to the thioredoxin superfamily, like glutaredoxins (GRXs) or thioredoxins (TRXs). These proteins participate as critical elements not only in the switch between the mitotic to the meiotic cycle but also at further developmental stages of microsporogenesis. They are also implicated in the regulation of pollen rejection as the result of self-incompatibility. In addition, they display precise space-temporal patterns of expression and are present in specific localizations like the stigmatic papillae or the mature pollen, although their functions and subcellular localizations are not clear yet. In this review we summarize insights and perspectives about the presence of thiol/disulphide-containing proteins in plant reproduction, taking into account the general context of the cell redox network.

## Introduction

Sexual Plant Reproduction involves complex biochemical pathways under a tight genetic control, which lead to drastic architectural changes at developmental and cellular levels (Franklin-Tong, 1999). These changes begin with the generation of the haploid gametes in specialized structures know as the mega- and microgametophytes: the embryo sac in the pistils and the pollen grain in the anthers. Afterwards, mature pollen grains land over a receptive stigma, hydrate and germinate, emerging a pollen tube, which enlarges at its apical region at exceptionally high growing rates. This pollen tube penetrates throughout the style toward the embryo sac in order to deliver the male gametes, which fertilize the egg cell and the polar nuclei generating an embryo and the endosperm, respectively, and ultimately the offspring (Sprunck et al., 2013).

Plants, like other eukaryotes, have evolved dedicating enormous resources and efforts to guarantee sexual reproduction. Among others, they have developed molecular mechanisms, which allow a tight regulation of all developmental events underlying the process. These mechanisms not only have contributed to assure the emergence of genetically-improved progenies, but also allowing plants to tune this process according to challenging environmental conditions, which has let flowering plants evolve dominating almost every terrestrial ecosystem (Hiscock, 2011). Redox regulation has recently emerged as a crucial mechanism able to manage significant stages during sexual plant reproduction where ROS, nitric oxide (NO) and other classical antioxidant protein and molecules are critically involved (Feijó et al., 2004; Prado et al., 2004; Dresselhaus and Franklin-Tong, 2013). In addition, several isoforms of plant redoxins like TRXs or GRXs seem to be specifically associated with reproductive tissues according to their precise space-temporal expression patterns (Table 1), although no clear functions have usually been assigned in this context.

**Table 1:**
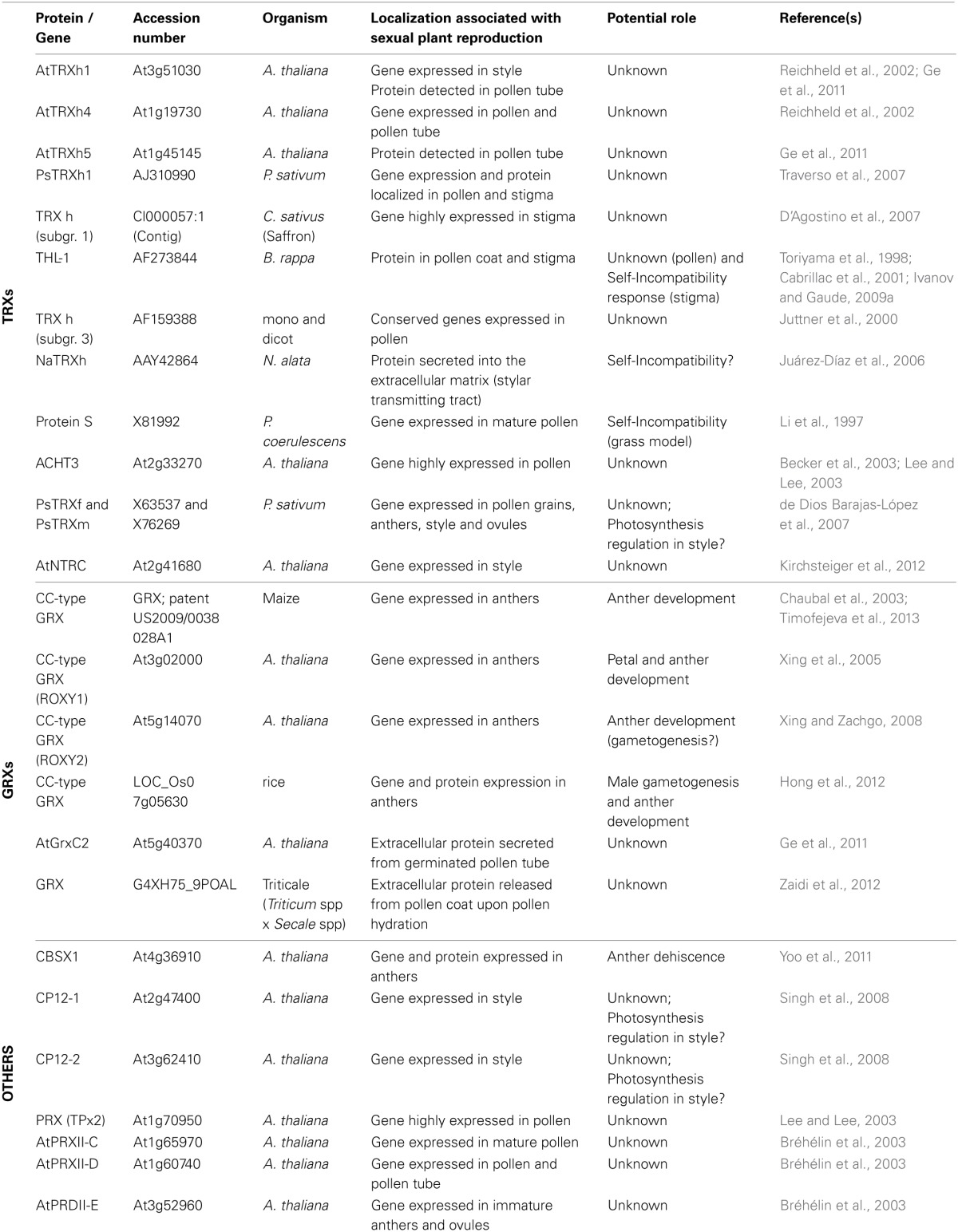
**Described redoxins and other related proteins involved in sexual plant reproduction**.

TRXs and GRXs are redox proteins whose activity depends on conserved cysteine (Cys) residues present in their active centers (Meyer et al., 2012). These Cys residues are usually maintained in their reduced state within the cell (Foyer and Noctor, 2005a). TRXs and GRXs carry out oxide-reductive reactions on essential Cys residues of a variety of plant proteins. The *Arabidopsis* genome contains about 40 genes encoding TRXs or TRX-related proteins grouped in different clusters and subclusters according to several aspects like sequence similarity, subcellular localization or intron position, which have been described as putatively involved in a plethora of plant roles (Buchanan and Balmer, 2005; Meyer et al., 2012). GRXs share important similarities with the TRX family like their protein structures (both protein types belong to the TRX superfamily) and the fact that higher plants also possess a larger number of GRX genes per genome when compared to other organisms (about 50 genes encoding for GRXs or GRX-related proteins in *Arabidopsis*) (Couturier et al., 2009; Meyer et al., 2012). The physiological roles of plant GRXs are less-known than those of TRXs, although they have also been involved in a variety of functions (Meyer et al., 2012).

Oxidized TRXs and GRXs are physiologically reduced by dedicated systems according to their subcellular localizations (for an extended review see Meyer et al., 2012). Cytosolic and mitochondrial TRXs are mainly reduced by NADPH in a reaction catalyzed by the enzyme NADPH-TRX-Reductase (NTR) (Arner and Holmgren, 2000; Laloi et al., 2001), while the isoforms from plastids are reduced by ferredoxin via the enzyme Ferredoxin-TRX-Reductase (FTR) (Shürmann and Jacquot, 2000; Balmer et al., 2006) and the GRXs are generally described as reduced by reduced glutathione (GSH). However, alternative crosstalk between both TRX and GRX systems has also been demonstrated in addition to these classical schemes, which reveals a high plasticity of the thiol-based redox regulation in plants (Gelhaye et al., 2003; Balmer et al., 2006; Reichheld et al., 2007; Bandyopadhyay et al., 2008; Marty et al., 2009).

TRXs and GRXs are described as the main protein families responsible for the redox status of protein Cys residues within the cell. These Cys are particularly susceptible to oxidations by reactive species, this fact being usually identified by researchers as a regulatory mechanism of the protein activity, as well as a protective or redox signaling mechanism (Couturier et al., 2013). These thiol-based regulations have been interpreted as a sensing mechanism of the cellular redox state, which acts between stress perception and plant response against environmental challenges (Foyer and Noctor, 2005a).

In this review we summarize, discuss and hypothesize about the occurrence of these thiol/disulfide containing proteins in reproductive tissues, pointing out an increased importance of the thiol-based redox regulation and signaling mechanisms in sexual plant reproduction.

### Redox regulation by ROS/RNS in sexual plant reproduction

Reactive species are produced in living cells as an unavoidable consequence of their own metabolism (Foyer and Noctor, 2005a). Apart from their activity damaging different macromolecules, they have been shown to act as secondary messengers, reason why the concept of oxidative stress has been re-evaluated (Foyer and Noctor, 2005b). Taking into account that protein Cys residues are particularly affected by reactive species, we review in this chapter the most important results involving ROS and RNS at different stages of the sexual plant reproduction.

**Figure 1:**
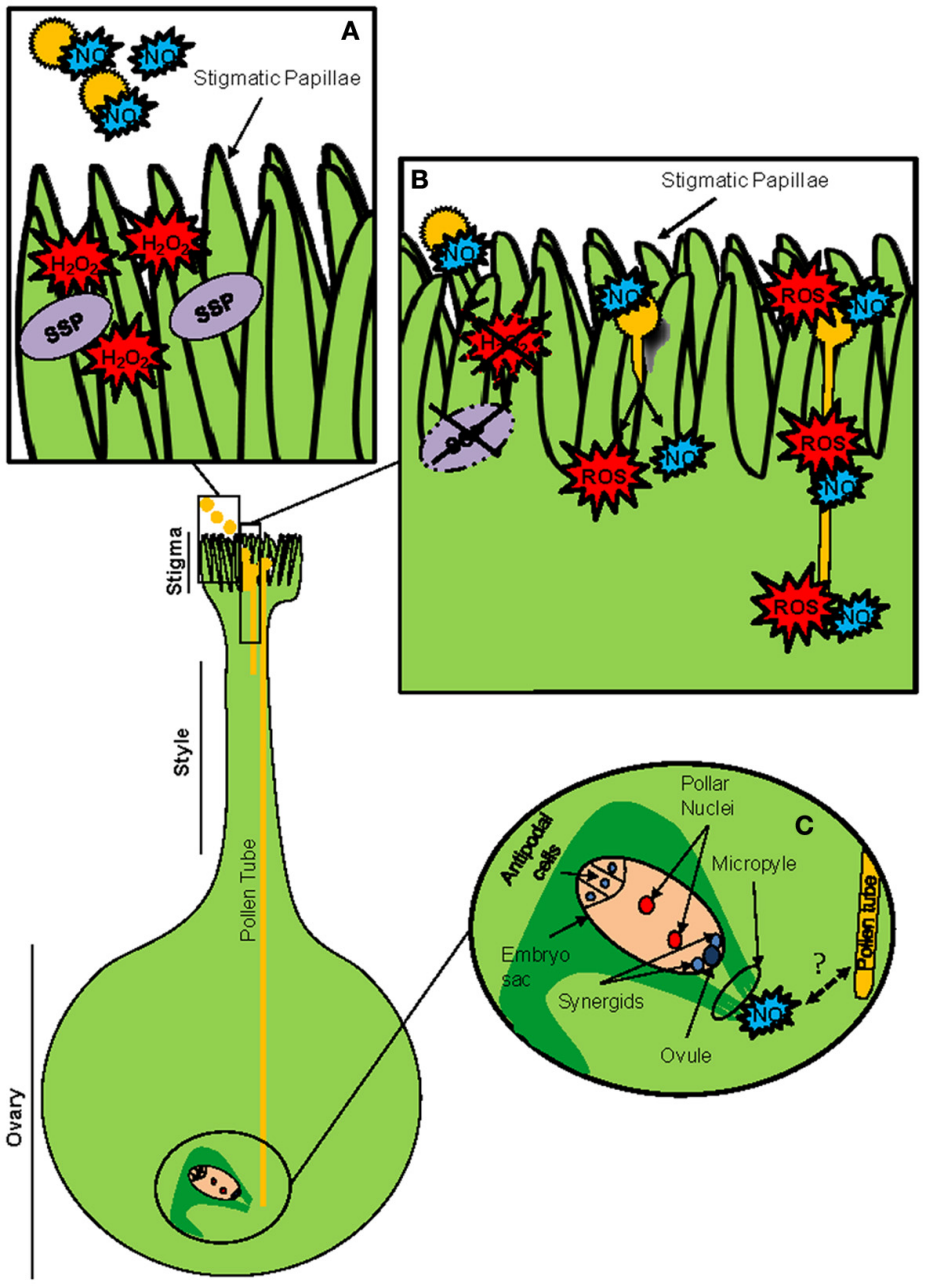
**ROS/NO are implicated in plant reproduction signaling.** Model gynoecium of an Angiosperm. **(A)** Receptive stigma at high magnification, before pollen-pistil interaction. Pollen grains are active NO producers, while the receptive stigma papillae generate large quantities of H_2_O_2_ and display enhanced peroxidase activity, in some cases in the form of stigma-specific peroxidase (SSP). **(B)** Stigma and pollen grains during diverse phases of their interaction. Both the enhanced peroxidase activity and the high levels of H_2_O_2_ become significantly reduced after pollen landing on the stigma and pollen germination, likely through pollen-produced NO signaling. The germinating pollen grains and the elongating pollen tubes produce ROS and NO, particularly at their tips. **(C)** The embryo sac is reached by the pollen tube tip trough the micropylar end. NO produced in micropyle could be involved in pollen tube guidance.

Reactive species have been shown to increase in a rapid and transient manner by specific molecular mechanisms for a proper plant growth and development, including sexual plant reproduction (Foreman et al., 2003; Potocký et al., 2007). ROS and NO have been involved in redox signaling taking place previously and during pollen-pistil interactions (Figure 1; Sharma and Bhatla, 2013). A high production of H_2_O_2_, exclusively confined to receptive stigmatic papillae was suggested to serve as a redox signal promoting pollen-pistil interaction and/or germination, as well as a defense mechanism against microbe attack (Figure 1A; McInnis et al., 2006a; Wilson et al., 2009; Zafra et al., 2010). In parallel to ROS production, a Stigma-Specific Peroxidase (SSP) was shown to be active only during a short period of stigma receptivity in *Senecio squalidus* (Figures 1A,B; McInnis et al., 2005, 2006b). *In vitro* experiments also revealed that mature pollen grains produce a high level of NO, which inhibits ROS production in the stigmatic papillae (Figure 1B; McInnis et al., 2006a; Bright et al., 2009; Zafra et al., 2010).

In addition, physiological mechanisms of ROS generation have been indicated to be required for normal pollen tube development (Figures 1B,C; Cardenas et al., 2006; Potocký et al., 2007, 2012). ROS production has been described to be mainly originated by the activity of specific isoforms of NADPH oxidases (NOX) localized at the tip of tobacco pollen tube (Figure 1B), whose activity was found to be increased by Ca^2+^ (Potocký et al., 2007). This represents a conserved mechanism of polarized cell tip growth (Bushart and Roux, 2007; Konrad et al., 2011).

In cucumber germinated pollen, ROS and NO production was specifically tip-localized during the initial germination steps, although was extended along the pollen tubes and the pollen grain later during germination (Figure 1B) (Šírová et al., 2011).

Both redox chemical agents and external conditions have shown to alter the production of these reactive species (Šírová et al., 2011). The addition of a reducer like ascorbate abolishes this production probably due to its capacity to effectively scavenge the intracellular ROS. The treatment with NO donors inhibits pollen germination and growth, and the addition of NO scavengers increases pollen germination rates (Prado et al., 2004; Šírová et al., 2011). NO seems also to be involved in the inhibition of germination and tube growth after pollen exposure to UV-B (Feng et al., 2000; He et al., 2007; Wang et al., 2010a,b). Curiously, NO exerts just the opposite effect in gymnosperm pollens, since it was shown that Pine pollen tube growth rate is accelerated by NO donors, whereas NO scavengers affect contrary (Wang et al., 2009a,b).

Although the mechanisms controlling pollen tube guidance and pollen-pistil interaction are still unknown (Boavida et al., 2005; Higashiyama and Hamamura, 2008; Márton and Dresselhaus, 2008) there is also evidence about the involvement of NO as a key molecule at this regard (Prado et al., 2004). Prado et al. (2008) showed that a Ca^2+^ specific response to NO induces pollen tube re-direction toward the ovule. Prado's work also describes the detection of NO production in the micropyle, where it was suggested to participate in pollen tube guidance to the ovules (Figure 1C; Prado et al., 2008). The generation of reactive oxygen species has also been involved in microsporogenesis, usually throughout programmed cell death (Jiang et al., 2007; Wan et al., 2007). In addition, it was recently shown that the male germ cell fate critically depends on H_2_O_2_ levels of the precursor cells (Kelliher and Walbot, 2012). Moreover, the molecular models developed in order to explain self-incompatibility (SI) in plants, usually include important roles for ROS or NO (McClure and Franklin-Tong, 2006; McInnis et al., 2006a,b; Wilkins et al., 2011).

Several of the detailed molecular mechanisms through which ROS and NO exert these functions are beginning to be outlined, and some of them involve thiol modifications. At this regard, Cys residues in proteins are particularly affected by these reactive species, and Cys-based signaling by ROS and/or RNS is a well-described feature affecting an increasing number of proteins, some of them from plants (Couturier et al., 2013; Corpas and Barroso, 2013). In bacteria, fungi or mammals, Cys modification by ROS and/or RNS has been described to affect DNA binding properties of some transcription factors (D'Autreaux and Toledano, 2007). In plant cells, the Cys-based actions of RNS (S-nitrosylation) and ROS (oxidation) on NPR1 and TGA1 proteins regulate plant systemic defense (Tada et al., 2008; Lindermayr et al., 2010). Protein S-nitrosylation is produced by the interaction of specific Cys residues with NO generated by different types of RNS, and this modification is emerging as a crucial regulatory mechanism involved in several aspect of plant physiology (Corpas et al., 2013). As an example, the activity of NADPH oxidases can be controlled by the S-nitrosylation of a C-terminal Cys (Yun et al., 2011). NOX proteins are highly involved in pollen tube growth and the subject of further regulation mechanisms (Potocký et al., 2007, 2012).

S-nitrosoglutathione (GSNO), an abundant molecule in plant tissues (Airaki et al., 2011) originated by S-nitrosylation of reduced glutathione (GSH) (Broniowska et al., 2013) is considered a reservoir, vehicle and biological donor of NO in plant cells (Corpas et al., 2013). Protein S-nitrosylation by GSNO (S-transnitrosation) also seems to be a feasible physiological mechanism for post-translational modification of proteins (Begara-Morales et al., 2013), however, not yet sufficiently described in plant reproductive tissues.

According to all these data, further experiments must be carried out to identify key proteins involved in the regulation of sexual plant reproduction via ROS or NO-mediated Cys oxidations. The specific presence of redoxins at these stages (Table 1; Figure 3) together with the importance of these reactive species suggests a more critical thiol-based regulation of several stages of the sexual plant reproduction than initially thought.

### Specific redoxins involved in anther development and male gametogenesis

**Figure 2:**
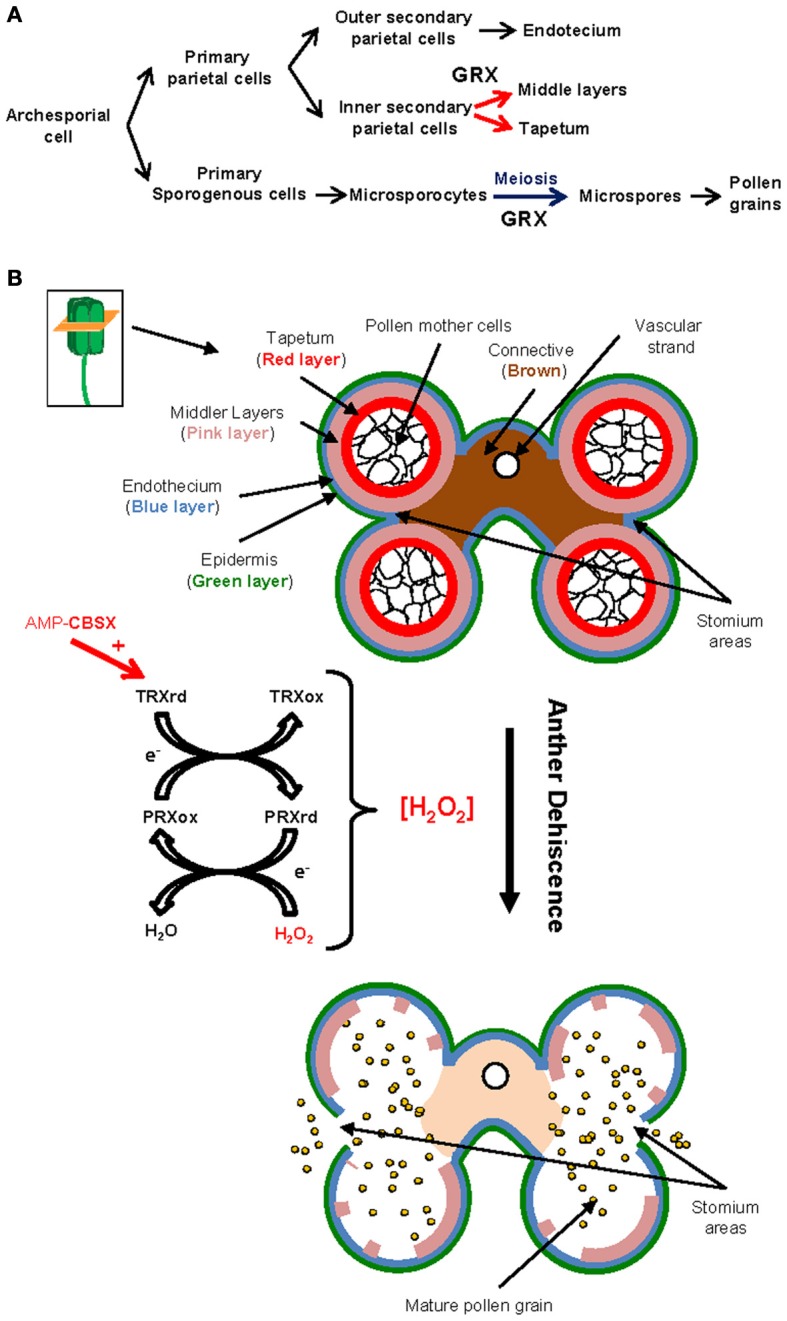
**Thiol-based redox proteins are critically involved in male gametogenesis, anther development and dehiscence. (A)** Ontogenic development of the male germline. CC-type GRXs are essential for the switch from mitosis to meiosis in the microsporocytes (blue arrow), which ultimately originate the haploid pollen grains. They also participate in the development of the anther layers surrounding the microsporocytes (red arrows). **(B)** Diagram representing the histological structure of an anther (transversal section) and major changes occurring after dehiscence. H_2_O_2_ is required for cell wall lignification, which induces a thickening of the endotecium leading to anther dehiscence. CBSXs can regulate the level of H_2_O_2_ via their ability to active TRXs, which ultimately reduce PRXs in the plastids. This activity is enhanced by the presence of AMP, thus connecting the nutrition state with anther development.

Successful sexual reproduction depends on the proper formation of specialized complex structures in the flower: anthers and pistils. Initially, a group of somatic cells must switch from the mitotic to the meiotic pathway to generate the haploid gametes. All processes are developed according to both environmental and developmental signals (Bhatt et al., 2001). Later, anther dehiscence will produce the release of mature pollen grains. Anther development and male gametogenesis processes are known to be critically influenced by the redox activity of specific thiol-based redox proteins. CC-type GRXs and the redox chloroplastidial system including CBSX (single cystathionine β-synthase domain–containing proteins)/TRX/PRX proteins play important roles in redox homeostasis and development in male reproductive tissues (Figure 2) (Wang et al., 2009a,b; Yoo et al., 2011).

CC-type GRXs, (also named ROXY proteins), are conserved plant-specific GRXs involved in anther and male gamete differentiation and flower development (Figure 2A; Xing et al., 2006; Wang et al., 2009a,b). The first evidence about such involvement was described by Chaubal et al. (2003) during the characterization of the maize mutant *msca1*. In this mutant, all anther cell layers were transformed into non-differentiated vegetative tissues. This phenotype was associated later with the lack of a GRX (Xing et al., 2011), and recently corroborated during the screening of male sterile lines in maize (Timofejeva et al., 2013). Culture of the *msca1* mutant under hypoxia conditions (low oxygen / H_2_O_2_) allows a rescue of the differentiation of the germinal line in the mutant flowers (Kelliher and Walbot, 2012).

However, probably the most important data concerning the role of ROXY proteins in anther development was provided by studies based on *A. thaliana*. Initially, the redox activity of the GRX ROXY1 was identified as a major regulator of early petal organ initiation and further steps of floral morphogenesis (Xing et al., 2005). Afterwards, the functionally redundant GRXs ROXY 1 and 2 were described to perform essential redox-dependent activities in early steps of anther and tapetum differentiation (Figure 2A; see anther structure in Figure 2B) by affecting the expression of a large variety of anther genes supporting critical roles (Xing and Zachgo, 2008). During anther development, they act via the redox activation of TGA9 and 10 transcription factors, probably among other protein targets (Murmu et al., 2010). *Arabidopsis* ROXY proteins were also suggested to be involved in male gametogenesis (Xing and Zachgo, 2008). In fact, this involvement has been recently evidenced in monocots (Hong et al., 2012). These authors have shown that the rice *MIL1* gene encodes for a CC-type GRX which is not only involved in the differentiation of the surrounding somatic layer of the anthers, but also in the switch of microsporocytes from mitosis to meiosis (Figure 2A). According to these results, pollen mother cells contain specific meiosis-initiation machinery in which this nuclear GRX (MIL1) plays preponderant roles, probably acting also via TGA-type transcription factors. In this context, the results from Kelliher and Walbot (2012), demonstrating that changes in the redox status critically control the male germ lineage fate in maize, suggest a master or integrator role of these types of GRXs in the redox regulation associated with anther and gamete differentiation.

In the anthers, the chloroplast redox system comprising CBSXs, TRXs and peroxiredoxins (PRXs) is involved in anther dehiscence and therefore pollen release via the control of H_2_O_2_ (Ok et al., 2012), which ultimately allows connecting plant nutritional information and pollen release (Figure 2B). CBSX are redox proteins characterized by sharing only a single pair of Cystathione β-Synthase domains (CBS) in their structures that belong to the CBS-containing protein (CDCPs) superfamily. *Arabidopsis* genome contains six genes encoding CBSX proteins (CBSX1-6), which have recently been described as cellular sensors involved in the control of plant redox homeostasis and development (Yoo et al., 2011). They act interacting and increasing the activity of TRXs by sensing cellular changes of adenosine nucleotides. CBSX1 is a member of this family in *Arabidopsis*, which is preferentially expressed in the chloroplast of the anther. This protein is able to interact and increase the activity of all four types of plastidial TRXs (*f*, *m x* and *y*). This augmentation is favored by the presence of AMP, but not by ADP or ATP (Figure 2B; Yoo et al., 2011; Ok et al., 2012). The overexpression of CBSX1 or CBSX2 in *Arabidopsis* transgenic plants yields plants showing a severe sterility as a consequence of the inhibition of their anther dehiscence, which prevents the liberation of mature pollen grains (Yoo et al., 2011; Jung et al., 2013). This sterility is due to a decrease of H_2_O_2_ in the anthers, which causes a lignin deficiency that originates a failure in the secondary wall thickening of the endothecium layer, and subsequently a very narrow crevice in the stomium area (region of the anther where dehiscence occurs and pollen grains leave the anthers; Figure 2B). Male sterility caused for this same reasons (a limitation of H_2_O_2_) have previously been reported (Karlsson et al., 2005; Villarreal et al., 2009). According to these authors, CBSX1 regulates the level of H_2_O_2_ via the activation of plastid TRXs, which reduce and activate peroxiredoxins (PRX) directly detoxifying this radical (Figure 2B). In our opinion, an evaluation of the roles of other enzymes or non-enzymatic systems known to be involved in the homeostasis of H_2_O_2_ would be of great interest at this regard. For example, the NADPH-TRX-Reductase C (NTRC) protein has been shown to be the main reducer of type-2 PRXs in chloroplasts (Kirchsteiger et al., 2009; Pulido et al., 2010). Apoplastic type III peroxidases (POXs), which are involved in cell wall polymerization via H_2_O_2_ regulation, should be assessed into their participation in this process (Hiraga et al., 2001). It is also well known the role of the ascorbic acid as main redox buffer in apoplast compartments (Foyer and Noctor, 2005a). Finally, overexpression of ROXY GRXs has also been shown to alter the level of H_2_O_2_ (Wang et al., 2009a,b).

Plant genomes contain higher number of genes encoding redoxins (like TRXs or GRXs) than other species, allowing them assigning specific isoforms to precise plant metabolic functions. This includes anther development and male gametogenesis. Remarkably, a similar case is found in mammals, where male germ cells are endowed with three testis-specific thioredoxins named Sptrx-1, Sptrx-2, and Sptrx-3, which are specially involved in spermatogenesis (Jiménez et al., 2004; Miranda-Vizuete et al., 2004).

### Unexpected and specific occurrence of redoxins in plant reproductive tissues suggest key functions in pollen-pistil interactions

**Figure 3:**
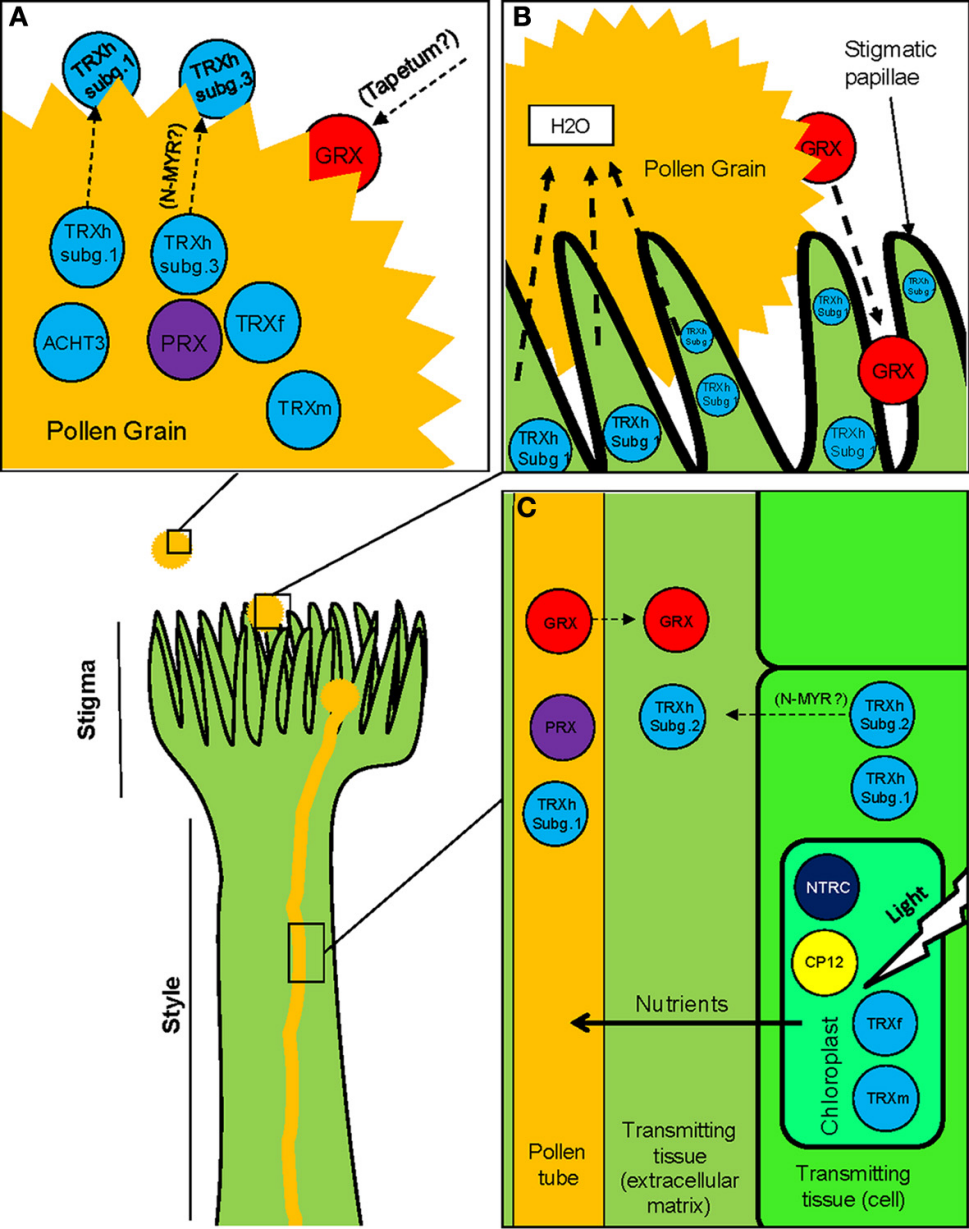
**Redoxins and related proteins are critical for pollen-pistil interactions.** Illustration of the stigma and style during pollen-pistil interaction. **(A)** Representation of a pollen grain and the pollen coat. The presence of redoxins has been described both within the pollen grain and in the pollen coat. It is particularly remarkable the specific and conserved occurrence of h-type TRXs in the pollen grains, which can be secreted to the pollen coat. We hypothesize that N-terminal lipidations may play a role in this mechanism of secretion. Also, some redoxins synthesized in the tapetum may become integrated into the pollen coat after tapetum degeneration **(B)** Image representing the initial stages of the pollen-stigma interaction. Upon pollen arrival, pollen starts to hydrate, rapidly releasing GRXs present in the pollen coat. Also, the stigmatic papillae are rich in subgroup 1 TRXs. These TRXs have been involved in SI processes in *Brassica* although their occurrence has also been described for self-compatible species. **(C)** Representation of a pollen tube growing throughout the transmitting tissues of the style. Different types of redoxins are present in the pollen tube, some of them being secreted to the extracellular matrix of the transmitting tissue. Other redoxins are mainly expressed in stylar cells and may also be secreted to the extracellular matrix. Several redoxins related to photosynthesis are specifically expressed in the cells of the stylar tissues, which suggest that high photosynthetic rates are probably supporting pollen tube growth.

Once a mature and dehydrated pollen grain lands on the appropriate stigma, it rapidly hydrates and incorporates nutrients from the stigma exudates (wet stigmas) or the stigma papillae (dry stigmas). This fact transforms the pollen grain into a polarized cell, which organizes its cytoplasm and cytoskeleton to support the extension of a tube within minutes after hydration (Edlund et al., 2004). This tube must grow to enter the transmitting tract along the style and finally reach the embryo sac where it will deliver the sperm nuclei that will participate of the double fertilization. At these stages, it is required a continuous interchange of both physical and chemical signals between partners (pollen, stigma, ovules…), which has to take place in a tight time frame (Dresselhaus and Franklin-Tong, 2013). Redox regulation and signaling by reactive species (Figure 1; see part 2) and probably by some redoxins (Table 1; Figure 3), might be critically involved in these signal exchanges.

#### Redoxins in pollen grain

TRXs *h* from subgroup 1 (Gelhaye et al., 2004), and GRXs are among the most abundant redoxins present in the pollen grain and the pollen coat (Figure 3). THL-1 is an h-type TRX that was immunodetected as a *B. rapa* extracellular pollen protein (Toriyama et al., 1998). However, although THL-1 is a well-known stigmatic protein involved in SI (Cabrillac et al., 2001), there is no clear data about its function in the pollen coat. The *Arabidopsis* h-type TRX*h*4 was shown to be expressed in the pollen grain and the pollen tube (Reichheld et al., 2002). Their counterparts AtTRX*h*1 and AtTRX*h*5, and the GRX AtGrxC2 proteins were detected in the pollen tube but not in the mature pollen grain. AtGrxC2 was also identified as a secreted protein (Figures 3A,C; Ge et al., 2011). Molecular and proteomic approaches also identified a GRX from triticale as a major pollen protein rapidly released upon pollen hydration on the stigma papillae (Figure 3B; Zaidi et al., 2012). This GRX contains a Gly in position 2, predicted to be co-translationally modified by N-myristoylation (N-MYR), a type of lipidation assisting protein anchoring to membranes, which therefore could account for secretion (Figure 3B; Denny et al., 2000; Utsumi et al., 2005; Martinez et al., 2008). The occurrence of this N-terminal modification has been recently evidenced *in vitro* for a similar GRX, AtGrxC1 from *Arabidopsis* (At5g63030) (Traverso, Pers. Commun.). It must be mentioned here that some of the proteins of the pollen coat are originated in the tapetum, and then incorporated into the pollen coat after the degeneration of this layer (Figure 2B), a mechanism that could be also suggested for the GRX released after hydration (Figures 2A,B; Zaidi et al., 2012).

The h-type TRXs belonging to the subgroup 3 (Gelhaye et al., 2004) were initially identified as a highly conserved group of pollen-expressed TRXs from both mono and dicots, featured by the presence of a N-terminal extension which contains conserved Gly and Cys residues in positions 2 and 4, respectively (Juttner et al., 2000). Curiously, these N-terminal extensions have been recently identified as a substrate for N-terminal myristoylation (N-MYR) as well as N-terminal palmitoylation (N-PAL). This last is another type of lipidation, usually identified in plasma membrane proteins (Traverso et al., 2008, 2013). No clear information is available concerning the specific roles or the subcellular localizations of these TRXs in pollen, although a member of this subgroup in *A. thaliana* (AtTRXh9) was shown to move from cell to cell via its N-terminal extension (Meng et al., 2010). According to these results, we hypothesized that these lipidations can be involved in the release of this subgroup of TRXs to the extracellular matrix, where others TRXs and redoxins have already been identified (Figure 3A; Ge et al., 2011; Zaidi et al., 2012).

Other types of redoxins have been identified as highly expressed in pollen. Two independent transcriptomic analyses (Becker et al., 2003; Lee and Lee, 2003) have shown ACHT3 TRX to be highly expressed in *Arabidopsis* pollen. However, no functional data are associated with this presence. In addition, PRXs are also among the redoxins displaying specific localization in pollen grains (Figure 3A). Transcriptomic analysis of *Arabidopsis* revealed two PRXs among the 50 most expressed genes in pollen. One of them (TPx2) showed increased expression under cold treatment (Lee and Lee, 2003). In addition, some type II PRXs from *Arabidopsis* showed specific expression patterns associated with male reproductive tissues (Figures 3A,C) (Bréhélin et al., 2003). The cytosolic AtPRXII-C is almost exclusively expressed in mature pollen, whereas its counterpart AtPRXII-D is detected in mature pollen, germinating pollen and pollen tubes, where both proteins could be reduced by AtTRXh4 (Reichheld et al., 2002; Bréhélin et al., 2003). Finally, the plastid-addressed AtPRDII-E has been described as mainly expressed in immature anthers and ovules (Bréhélin et al., 2003).

Considering that no clear functional data are associated with this specific occurrence in pollen grains, further work is necessary to better understand the precise redox mechanisms underlying pollen function. It is well-known that when pollen grains reach the appropriated stigma, they release a number of proteins from pollen coat, which together with the proteins released from pistil surrounding tissues, seem to play important roles during pollen-pistil interaction, adhesion, germination or pollen tube growth as well as providing protection against pathogen attack (Andersson and Lidholm, 2003; Grote et al., 2008; Zaidi et al., 2012). In a different context, redoxins from the pollen coat have been attributed with some allergic potential (Toriyama et al., 1998). However, there is no evidence supporting this fact, with the exception of the description of a 1-Cys PRXs and a h-type TRX as respiratory wheat flours allergens from maize (Fasoli et al., 2009; Pahr et al., 2012).

#### Occurrence of GR/GRX and NTR/TRX systems during pollen germination and pollen tube growth

Several works based on the characterization of *A. thaliana* mutant lines have evidenced that GRXs and TRXs are specifically involved in pollen germination and pollen tube growth. The *Arabidopsis* double mutant *ntra ntrb*, lacking NTR activity to reduce cytosolic h-type TRXs, showed a reduced fitness due to defects in pollen functions (Reichheld et al., 2007). Under this genetic background, GRXs are also able of directly reduce h-type TRXs although in lesser extension, thus revealing a more complex *in vivo* interplay between the TRX and glutathione pathways (Reichheld et al., 2007). In fact, the additional disruption of the glutathione reductase 1 gene (*GR1*) under this double mutant background (triple mutant *ntra ntrb gr1*) led to a pollen lethal phenotype (Marty et al., 2009). Noteworthy, the characterization of the single mutant *gr1* demonstrated that a residual reduction of GSSG could be directly attributed to h-type TRXs, reason why this NTR/TRX system was described as a functional backup for the activity of GR1 (Marty et al., 2009).

The characterizations of the double (*ntra ntrb*) and triple mutant (*ntra ntrb gr1*) headed to other important conclusions. Defects in pollen grains were not associated with gametogenesis, since mature pollen grains inside the anthers were viable in both mutants (Reichheld et al., 2007; Marty et al., 2009). Then it is important to elucidate, in our opinion, in which subsequent process these redox systems are critically involved: (i) pollen germination, (ii) pollen tube growth, (iii) polarity or guidance, or (iv) pollen tube–embryo sac interactions and fertilization.

A second conclusion is that the lack of both redox systems drastically affected male gametophyte functions, contrary to what occurred in the female haploid gametophyte. The unpaired NTR-TRX System (NTS) in the double *ntra ntrb* mutant yielded reduced fitness in pollen grains, probably derived from a limited reduction of h-type TRXs, although the diploid sporophyte or the female gametophyte did not show such drastic phenotypes (Reichheld et al., 2007). In a similar way, the *ntra ntrb gr1* triple mutant produced a lethal phenotype in pollen, while the female embryo sac was unaffected (Marty et al., 2009). This strong difference is probably associated with the exceptional burst metabolism occurring during pollen germination, that produces important redox imbalances and would justify the exceptional requirement of both thiol-dependent redox GR/GRX and NTR/TRX systems. This is in agreement with the results compiled in our present review, showing how the h-type TRXs or the GRXs are specifically found in pollen grain, pollen tube or pollen coat.

Within this context, it must be noted the importance of GSH and auxins for pollen functionality. Zechmann et al. (2011) have shown that GSH availability is essential for pollen germination and early elongation steps of the pollen tube, since its depletion triggers disturbances in the auxin metabolism, which led to inhibition of pollen germination. Considering that NTR/TRX and GSH pathways are involved in auxin homeostasis (Bashandy et al., 2010, 2011), we suggest that both redox systems can act somehow linking the GSH cellular status and auxin downstream signals during and/or after pollen germination.

#### Redoxins in female reproductive tissues

TRXs h have been shown to be specifically expressed in the stigmatic papillae of different plants (Figure 3B). The PsTRXh1 from *P. sativum* has been immunolocalized in the receptive stigmatic papillae and the mature pollen grain of this plant (Traverso et al., 2007). THL-1 from *B. rapa*, a TRXs h from the same subgroup, which has been involved in SI (Cabrillac et al., 2001) also shows a similar pattern of expression in flower tissues than PsTRXh1. Considering that *P. sativum* is a self-compatible species, the role associated with this dual localization in pea flower is unlikely implicated in SI. Besides, an h-type TRX from subgroup I was included within the 50 most highly expressed genes characterized in a transcriptomic analysis from saffron stigmas (D'Agostino et al., 2007), and another TRX, h-type *Arabidopsis* h1 was distinctively detected in the style (Reichheld et al., 2002).

Classical chloroplastidial TRXs or TRX-related proteins have also been associated with pistils (Figure 3C). For example, expression of Pea TRXs m and f types was specifically detected in pollen grains, tapetum, style and ovules (de Dios Barajas-López et al., 2007). Other plastidial TRX-related proteins like the NTRC or CP12 redox proteins are also present in the style (Figure 3C). CP12 are small, dithiol-based redox-sensitive proteins which together with the plastidial TRX f, regulate the activity of the Calvin cycle in response to rapid light changes (Howard et al., 2008). The redox state of CP12 has been shown to be regulated by TRXs (Marri et al., 2009). The *Arabidopsis* genome contains three genes encoding CP12 proteins (CP12-1, 2 and 3), which could be involved in plant reproduction since CP12-1 and 2 are specifically expressed in the style (Singh et al., 2008). No clear roles are associated with these expressions, although CP12-antisense lines of tobacco and *Arabidopsis* display a complex phenotype including reduced fertility (Singh et al., 2008). AtNTRC is also a redox plastidial protein characterized as the principal reducer of 2-Cys PRXs (Kirchsteiger et al., 2009; Pulido et al., 2010). This protein allows the use of NADPH to maintain the redox status of the chloroplast (Spínola et al., 2008), as well as acting as a general molecular switch able to convert NADPH into redox signal in non-photosynthetic plastids (Kirchsteiger et al., 2012). Remarkably, this protein also shows a high level of expression in the style (Kirchsteiger et al., 2012).

Pollen tube growth throughout the style depends on nutrients supported by female tissues (Lind et al., 1996; Taylor and Hepler, 1997; Wu et al., 2001). The presence of photosynthesis regulatory proteins like those described here might be derived from the necessity of extra nutrients from the stylar photosynthetic cell to feed pollen tubes. However, we cannot exclude other possibilities like their involvement in the control of the redox imbalance derived from the burst metabolism of the growing pollen tube. Under this context, the redox state of two key enzymes of the Benson-Calvin cycle (fructose-1,6-bisphosphatase and phosphoribulokinase) could be regulated by S-nitrosylation, since they have been previously identified as S-nitrosylated proteins (Lindermayr et al., 2005; Begara-Morales et al., 2013).

### Self-incompatibility and h-type TRXs

Self-incompatibility (SI) is a mechanism adopted by flowering plants to prevent self-fertilization as well as to avoid crossings with genetically-related plants, promoting outcrossing and subsequently, genetic diversity in the offspring (Iwano and Takayama, 2012). Therefore, this mechanism has been critically relevant on the dominant position reached by flowering plants in the biosphere during evolution (Gaude and Cabrillac, 2001). Genes involved in SI display high allelic variability, and are referred as haplotypes in the literature. SI systems are based on the discrimination between male-specificity and female-specificity determinants (S-determinants) localized in the surface of pollen grains and the stigmatic papillae respectively, both encoded at the S-locus. Therefore, SI depends on a specific interaction between male and female S-determinants derived from the same or different S-haplotypes (depending on the plant family. For a review see Iwano and Takayama, 2012). Although these determinants differ among plant species, the redox activity of the TRXs h has been described as critically involved within most of the proposed SI molecular models.

In the plant *Phalaris coerulescens*, the SI model system is based on two multiallelic not-linked loci, called S and Z (Hayman and Richter, 1992; Li et al., 1997; Klaas et al., 2011). Locus S encodes a monocot-conserved protein exclusively expressed in mature pollen. Its N-terminal domain is highly variable and contains the allelic information, whereas the C-terminal end contains a conserved TRX h-type motif responsible of the catalytic activity and its disruption yields the lost of the SI (Li et al., 1994, 1995). Although no enough data are available for assembling a clear model, the redox activity of the TRX domain has been shown to be essential for the SI response in *P. coerulescens* (Li et al., 1996).

In the classical model of *Brassica*, several genes are involved in SI, all of them linked to the S-locus. Two of these genes encode for the female and male determinants, the S-locus Receptor Kinase (SRK) expressed in the stigma-papilla cells and its ligand, the S-locus Cys rich protein (SCR) localized in the pollen coat (Schopfer et al., 1999; Takasaki et al., 2000). Pollen rejection is achieved when SRK recognizes SCR coming from the same S-allele. SRK is a transmembrane protein with a serine threonine kinase activity in the cytoplasmic region, which triggers the downstream biochemical pathways of the SI response (Giranton et al., 2000). In a classical model suggested by Cabrillac et al. (2001), this kinase activity is reversibly blocked by the direct interaction with a h-type TRX (THL-1) in the subcortical cytoplasmic side of stigma papillae cells, and only the allele-specific interaction between SCR and SRK produces the release of the TRX, therefore releasing the kinase activity (Cabrillac et al., 2001). However, according to Ivanov and Gaude (2009a), SRK is mostly intracellular, and the scarce part present in different domains of the plasma membrane can interact with SCR. After ligand recognition, the receptor-ligand complex is internalized in endosomes enriched in the negative regulator TRX h (Ivanov and Gaude, 2009a). In fact, these authors proposed a new model, the SI “domain” model, where the role of the TRX is mainly relegated to the endomembrane system (Ivanov and Gaude, 2009b). h-type TRXs are the biggest cluster of plant TRXs, encoded by almost eleven TRXs in the genome of *A. thaliana*, which have been usually described as soluble proteins due to the lack of transit peptides (Florencio et al., 1988). However, some TRXs h have recently been described to be modified by lipidation (myristoylation and palmitoylation) at their N-terminal extensions. These modifications result in their localization to the endomembrane system or to the plasma membrane (Traverso et al., 2008, 2013) and are in agreement with the endomembrane localizations of the TRXs h shown by Ivanov and Gaude (2009a). Independently from the models suggested, the role of the TRX h in *Brassica* SI mechanism was demonstrated, since antisense transgenic lines for THL-1 displayed a limited level of SI only (Haffani et al., 2004). Furthermore, it has been shown that this role depends on both the redox activity of the TRX, and the occurrence of a specific sequence in their active centers -WCPPC- (Mazzurco et al., 2001).

The SI model mechanism described for *Solanaceae, Rosaceae, and Plantaginaceae* families is based on the activity of secreted S-RNases. In all three families, compatibility is also controlled by a polymorphic S-locus containing at least two genes. S-RNases determine the specificity of pollen rejection in the pistil, and S-locus F-box proteins fulfill this function in pollen. In *N. alata*, Juárez-Díaz et al. (2006) suggested the participation of a h-type TRX (NaTRXh), which interacts with the S-RNase in the extracellular matrix of the stylar transmitting tract (Figure 3C). Although these authors suggested that NaTRXh was secreted due to some information present in its N-terminal extension, the secretion mechanism remained unexplained. Remarkably, NaTRXh has also been characterized as a N-myristoylated membrane-associated protein. Therefore, this characteristic could be involved in its secretion (Figure 3C; Traverso et al., 2013).

ROS and RNS have also been shown to be involved in SI mechanisms. In the *Papaver* model of SI (where no TRXs have been directly implicated), SI response seems to be mediated by ROS/NO redox signaling. Allele-specific interaction was shown to induce a rapid and transitory increase of ROS and NO in *Papaver rhoeas* pollen tubes. In this model, SI is triggered by an increase of intracellular Ca^2+^ in the pollen tube, which ultimately originates actin reorganization and programmed cell death resulting in the destruction of the self-pollen. ROS/NO seem to act mediating the signal between calcium and PCD (Wilkins et al., 2011). In the S-RNase-based model of SI response, the S-RNase specifically disrupts tip-localized ROS of incompatible pollen tubes via arresting ROS formation in mitochondria and cell walls of *Pyrus pyrifolia* (Wang et al., 2010a,b).

All these SI mechanisms usually originate the rejection of the allele-incompatible pollen grains by affecting the pollen tube growth throughout the stigma and the style. Although independent SI molecular mechanisms are known, which indicates that SI has clearly evolved independently several times during plant evolution (Takayama and Isogai, 2005), the involvement of the TRXs (and probably other redoxins) as well as the signaling mediated by ROS or NO have been evidenced in different SI models proposed. Further experiments are needed to clarify the role of redoxins in all these models within the general context of redox regulation and signaling, which includes reactive species-mediated signaling.

## Concluding remarks

In this review, we have listed and discussed the most remarkable evidences suggesting that molecular and cellular events involved in sexual plant reproduction are critically influenced by plant-conserved and specific thiol-based redox mechanisms, which include ROS and NO together with several isoforms of TRXs, GRXs and other redox-related proteins. These plant reproductive-specific redox mechanisms likely appeared as a consequence of the high number of isoforms of redoxins available, which emerged during plant evolution and somehow resulted in beneficial characteristic for plant physiology. However, and comparatively with non-reproductive plant tissues and organs, much is still unknown about the nature, presence, localization and molecular features of these proteins.

### Conflict of interest statement

The authors declare that the research was conducted in the absence of any commercial or financial relationships that could be construed as a potential conflict of interest.

## References

[B1] Airaki M. Sánchez-Moreno L. Leterrier M. Barroso J. B. Palma J. M. Corpas F. J. (2011). Detection and quantification of S-nitrosoglutathione (GSNO) in pepper (*Capsicum annuum* L.) plant organs by LC-ES/MS. Plant Cell Physiol. 52, 2006–2015 10.1093/pcp/pcr133 21965607

[B2] Andersson K. Lidholm J. (2003). Characteristics and immunobiology of grass pollen allergens. Int. Arch. Allergy Immunol. 130, 87–107 10.1159/000069013 12673063

[B3] Arner E. S. J. Holmgren A. (2000). Physiological functions of thioredoxin and thioredoxin reductase. Eur. J. Biochem. 267, 6102–6109 10.1046/j.1432-1327.2000.01701.x 11012661

[B4] Balmer Y. Vensel W. H. Cai N. Manieri W. Schürmann P. Hurkman W. J. (2006). A complete ferredoxin/thioredoxin system regulates fundamental processes in amyloplasts. Proc. Natl. Acad. Sci. U.S.A. 103, 2988–2993 10.1073/pnas.0511040103 16481623 PMC1413819

[B5] Bandyopadhyay S. Gama F. Molina-Navarro M. M. Gualberto J. M. Claxton R. Naik S. G. (2008). Chloroplast monothiol glutaredoxins as scaffold proteins for the assembly and delivery of [2Fe-2S] clusters. EMBO J. 27, 1122–1133 10.1038/emboj.2008.50 18354500 PMC2323258

[B6] Bashandy T. Guilleminot J. Vernoux T. Caparros-Ruiz D. Ljung K. Meyer Y. (2010). Interplay between the NADP-linked thioredoxin and glutathione systems in *Arabidopsis* auxin signaling. Plant Cell 22, 376–391 10.1105/tpc.109.071225 20164444 PMC2845418

[B7] Bashandy T. Meyer Y. Reichheld J. (2011). Redox regulation of auxin signaling and plant development in *Arabidopsis*. Plant Signal. Behav. 6, 117–119 10.4161/psb.6.1.14203 21422826 PMC3122021

[B8] Becker J. D. Boavida L. C. Carneiro J. Haury M. Feijó J. A. (2003). Transcriptional profiling of *Arabidopsis* tissues reveals the unique characteristics of the pollen transcriptome. Plant Physiol. 133, 713–725 10.1104/pp.103.028241 14500793 PMC219046

[B9] Begara-Morales J. C. López-Jaramillo F. J. Sánchez-Calvo B. Carreras A. Ortega-Muñoz M. Santoyo-González F. (2013). Vinyl sulfone silica: application of an open preactivated support to the study of transnitrosylation of plant proteins by S-nitrosoglutathione. BMC Plant Biol. 12, 13–61 . 10.1186/1471-2229-13-61 23586608 PMC3639107

[B10] Bhatt A. M. Canales C. Dickinson H. G. (2001). Plant meiosis: the means to 1N. Trends Plant. Sci. 6, 114–121 10.1016/S1360-1385(00)01861-6 11239610

[B11] Boavida L. Becker J. D. Vieira A. M. Feijó J. A. (2005). Gametophyte interaction and sexual reproduction: how plants make a zygote. Int. J. Dev. Biol. 49, 615–632 10.1387/ijdb.052023lb 16096969

[B12] Bréhélin C. Meyer E. H. de Souris J. P. Bonnard G. Meyer Y. (2003). Resemblance and dissemblance of *Arabidopsis* type II peroxiredoxins: similar sequences for divergent gene expression, protein localization, and activity. Plant Physiol. 132, 2045–2057 10.1104/pp.103.022533 12913160 PMC181289

[B13] Bright J. Hiscock S. J. James P. E. Hancock J. T. (2009). Pollen generates nitric oxide and nitrite: a possible link to pollen-induced allergic responses. Plant Physiol. Biochem. 47, 49–55 10.1016/j.plaphy.2008.09.005 18964065

[B14] Broniowska K. A. Diers A. R. Hogg N. (2013). S-Nitrosoglutathione. Biochim. Biophys. Acta 1830, 3173–3181 10.1016/j.bbagen.2013.02.004 23416062 PMC3679660

[B15] Buchanan B. B. Balmer Y. (2005). Redox regulation: a broadening horizon. Annu. Rev. Plant Biol. 56, 187–220 10.1146/annurev.arplant.56.032604.144246 15862094

[B16a] Bushart T. J. Roux S. J. (2007). Conserved features of germination and polarized cell growth: a few insights from a pollen-fern spore comparison. Ann. Bot. 99, 9–17 10.1093/aob/mcl159 16867999 PMC2802967

[B16] Cabrillac D. Cock J. M. Dumas C. Gaude T. (2001). The S-locus receptor kinase is inhibited by thioredoxins and activated by pollen coat proteins. Nature 410, 220–223 10.1038/35065626 11242083

[B17] Cardenas L. McKenna S. T. Kunkel J. G. Hepler P. K. (2006). NAD(P)H oscillates in pollen tubes and is correlated with tip growth. Plant Physiol. 142, 1460–1468 10.1104/pp.106.087882 17041030 PMC1676060

[B18] Chaubal R. Anderson J. R. Trimnell M. R. Fox T. W. Albertsen M. C. Bedinger P. (2003). The transformation of anthers in the msca1 mutant of maize. Planta 216, 778–788 10.1007/s00425-002-0929-8 12624765

[B19] Corpas F. J. Alché J. D. Barroso J. B. (2013). Current overview of S-nitrosoglutathione (GSNO) in higher plants. Front. Plant Sci. 4:126 10.3389/fpls.2013.00126 23658557 PMC3647110

[B20] Corpas F. J. Barroso J. B. (2013). Nitro-oxidative stress vs oxidative or nitrosative stress in higher plants. New Phytol. 199, 633–635 10.1111/nph.12380 23763656

[B21] Couturier J. Chibani K. Jacquot J. P. Rouhier N. (2013). Cysteine-based redox regulation and signaling in plants. Front. Plant Sci. 4:105 10.3389/fpls.2013.00105 23641245 PMC3638127

[B22] Couturier J. Jacquot J. P. Rouhier N. (2009). Evolution and diversity of glutaredoxins in photosynthetic organisms. Cell Mol. Life Sci. 66, 2539–2557 10.1007/s00018-009-0054-y 19506802 PMC11115520

[B23] D'Autreaux B. Toledano M. B. (2007). ROS as signalling molecules: mechanisms that generate specificity in ROS homeostasis. Nat. Rev. Mol. Cell Biol. 8, 813–824 10.1038/nrm2256 17848967

[B24] D'Agostino N. Pizzichini D. Chiusano M. L. Giuliano G. (2007). An EST database from saffron stigmas. BMC Plant Biology 7:53 10.1186/1471-2229-7-53 17925031 PMC2221943

[B25] de Dios Barajas-López J. Serrato A. J. Olmedilla A. Chueca A. Sahrawy M. (2007). Localization in roots and flowers of pea chloroplastic thioredoxin f and thioredoxin m proteins reveals new roles in nonphotosynthetic organs. Plant Physiol. 145, 946–960 10.1104/pp.107.105593 17885084 PMC2048802

[B26] Denny P. W. Gokool S. Russell D. G. Field M. C. Smith D. F. (2000). Acylation-dependent protein export in Leishmania. J. Biol. Chem. 275, 11017–11025 10.1074/jbc.275.15.11017 10753904

[B27] Dresselhaus T. Franklin-Tong N. (2013). Male-female crosstalk during pollen germination, tube growth and guidance, and double fertilization. Mol. Plant 6, 1018–1036 10.1093/mp/sst061 23571489

[B28] Edlund A. F. Swanson R. Preuss D. (2004). Pollen and stigma structure and function: the role of diversity in pollination. Plant Cell 16, S84–97 10.1105/tpc.015800 15075396 PMC2643401

[B29] Fasoli E. Pastorello E. A. Farioli L. Scibilia J. Aldini G. Carini M. (2009). Searching for allergens in maize kernels via proteomic tools. J. Proteomics 13, 501–510 10.1016/j.jprot.2009.01.013 19367736

[B30] Feijó J. A. Costa S. S. Prado A. M. Becker J. D. Certal A. C. (2004). Signalling by tips. Curr. Opin. Plant Biol. 7, 589–598 10.1016/j.pbi.2004.07.014 15337103

[B31] Feng H. Lizhe A. Tan L. Hou Z. Wang X. (2000). Effect of enhanced ultraviolet-B radiation on pollen germination and tube growth of 19 taxa *in vitro*. Environ. Exp. Bot. 43, 45–53 10.1016/S0098-8472(99)00042-812385209

[B32] Florencio F. J. Yee B. C. Johnson T. C. Buchanan B. B. (1988). An NADP/thioredoxin system in leaves: purification and characterization of NADP-thioredoxin reductase and thioredoxin h from spinach. Arch. Biochem. Biophys. 266, 496–507 10.1016/0003-9861(88)90282-2 3190242

[B33] Foreman J. Demidchik V. Bothwell J. H. Mylona P. Miedema H. Torres M. A. (2003). Reactive oxygen species produced by NADPH oxidase regulate plant cell growth. Nature 27, 442–446 10.1038/nature01485 12660786

[B34] Foyer C. H. Noctor G. (2005a). Redox homeostasis and antioxidant signaling. A metabolic interface between stress perception and physiological responses. Plant Cell 17, 1866–1875 10.1105/tpc.105.033589 15987996 PMC1167537

[B35] Foyer C. H. Noctor G. (2005b). Oxidant and antioxidant signalling in plants: A reevaluation of the concept of oxidative stress in a physiological context. Plant Cell Environ. 28, 1056–1071 10.1111/j.1365-3040.2005.01327.x

[B36] Franklin-Tong V. E. (1999). Signaling and the modulation of pollen tube growth. Plant Cell 11, 727–738 10213789 10.1105/tpc.11.4.727 PMC144203

[B37] Gaude T. Cabrillac D. (2001). Self-incompatibility in flowering plants: the Brassica model. C. R. Acad. Sci. III 324, 537–542 10.1016/S0764-4469(01)01323-3 11455876

[B38] Ge W. Song Y. Zhang C. Zhang Y. Burlingame A. L. Guo Y. (2011). Proteomic analyses of apoplastic proteins from germinating *Arabidopsis thaliana* pollen. Biochem. Biophys. Acta 1814, 1964–1973 10.1016/j.bbapap.2011.07.013 21798377 PMC3214693

[B39] Gelhaye E. Rouhier N. Jacquot J. P. (2003). Evidence for a subgroup of thioredoxin h that requires GSH/Grx for its reduction. FEBS Lett. 555, 443–448 10.1016/S0014-5793(03)01301-2 14675753

[B40] Gelhaye E. Rouhier N. Jacquot J. P. (2004). The thioredoxin h system of higher plants. Plant Physiol. Biochem. 42, 265–271 10.1016/j.plaphy.2004.03.002 15120110

[B41] Giranton J. L. Dumas C. Cock J. M. Gaude T. (2000). The integral membrane S-locus receptor kinase of Brassica has serine/threonine kinase activity in a membranous environment and spontaneously forms oligomers in planta. Proc. Natl. Acad. Sci. U.S.A. 97, 3759–3764 10.1073/pnas.97.7.3759 10725390 PMC16313

[B42] Grote M. Westritschnig K. Valenta R. (2008). Immunogold electron microscopic localization of the 2 EF-hand calcium-binding pollen allergen Phl p 7 and its homologues in pollens of grasses, weeds and trees. Int. Arch. Allergy Immunol. 146, 113–121 10.1159/000113514 18204277

[B43] Haffani Y. Z. Gaude T. Cock J. M. Goring D. R. (2004). Antisense suppression of thioredoxin h mRNA in Brassica napus cv. Westar pistils causes a low level constitutive pollen rejection response. Plant Mol. Biol. 55, 619–630 10.1007/s11103-004-1126-x 15604705

[B44] Hayman D. L. Richter J. (1992). Mutations affecting self-incompatibility in Phalaris-Coerulescens Desf (Poaceae). Heredity 6, 8495–8503

[B45] He J. M. Bai X. L. Wang R. B. Cao B. She X. P. (2007). The involvement of nitric oxide in ultraviolet-B-inhibited pollen germination and tube growth of Paulownia tomentosa *in vitro*. Physiol. Plant. 131, 273–282 18251898 10.1111/j.1399-3054.2007.00955.x

[B46] Higashiyama T. Hamamura Y. (2008). Gametophytic pollen tube guidance. Sex. Plant Reprod. 21, 17–26 10.1007/s00497-007-0064-6

[B47] Hiraga S. Sasaki K. Ito H. Ohashi Y. Matsui H. (2001). A large family of class III plant peroxidases. Plant Cell Physiol. 42, 462–468 10.1093/pcp/pce061 11382811

[B48] Hiscock S. J. (2011). Sexual plant reproduction. Ann. Bot. 108, 585–587 10.1093/aob/mcr217 22010286 PMC3170161

[B49] Hong L. Tang D. Zhu K. Wang K. Li M. Cheng Z. (2012). Somatic and reproductive cell development in rice anther is regulated by a putative glutaredoxin. Plant Cell 24, 577–588 10.1105/tpc.111.093740 22319054 PMC3315234

[B50] Howard T. P. Metodiev M. Lloyd J. C. Raines C. A. (2008). Thioredoxin-mediated reversible dissociation of a stromal multiprotein complex in response to changes in light availability. Proc. Natl. Acad. Sci. U.S.A. 105, 4056–4061 10.1073/pnas.0710518105 18322016 PMC2268787

[B51] Ivanov R. Gaude T. (2009a). Endocytosis and endosomal regulation of the S-receptor kinase during the self-incompatibility response in Brassica oleracea. Plant Cell 21, 2107–2017 10.1105/tpc.108.063479 19622804 PMC2729615

[B52] Ivanov R. Gaude T. (2009b). Brassica self-incompatibility: a glimpse below the surface. Plant Signal. Behav. 4, 996–998 10.4161/psb.4.10.9714 19826214 PMC2801372

[B53] Iwano M. Takayama S. (2012). Self/non-self discrimination in angiosperm self-incompatibility. Curr. Opin. Plant Biol. 15, 78–83 10.1016/j.pbi.2011.09.003 21968124

[B54] Jiang P. Zhang X. Zhu Y. Zhu W. Xie H. Wang X. (2007). Metabolism of reactive oxygen species in cotton cytoplasmic male sterility and its restoration. Plant Cell Rep. 26, 1627–1634 10.1007/s00299-007-0351-6 17426978

[B55] Jiménez A. Zu W. Rawe V. Y. Pelto-Huikko M. Flickinger C. J. Sutovsky P. (2004). Spermatocyte/spermatid-specific thioredoxin-3, a novel Golgi apparatus-associated thioredoxin, is a specific marker of aberrant spermatogenesis. J. Biol. Chem. 279, 34971–34982 10.1074/jbc.M404192200 15181017

[B56a] Juárez-Díaz J. A. McClure B. Vázquez-Santana S. Guevara-García A. León-Mejía P. Márquez-Guzmán J. (2006). A novel thioredoxin h is secreted in Nicotiana alata and reduces S-RNase in vitro. J. Biol. Chem. 281, 3418–3424 10.1074/jbc.M511687200 16354655

[B56] Jung K. W. Kim Y. Y. Yoo K. S. Ok S. H. Cui M. H. Jeong B. C. (2013). A Cystathionine-β-synthase domain-containing protein, CBSX2, regulates endothecial secondary cell wall thickening in anther development. Plant Cell Physiol. 54, 195–208 10.1093/pcp/pcs166 23220733

[B57] Juttner J. Olde D. Langridge P. Baumann U. (2000). Cloning and expression of a distinct subclass of plant thioredoxins. Eur. J. Biochem. 267, 7109–7117 10.1046/j.1432-1327.2000.01811.x 11106422

[B58] Karlsson M. Melzer M. Prokhorenko I. Johansson T. Wingsle G. (2005). Hydrogen peroxide and expression of hipI-superoxide dismutase are associated with the development of secondary cell walls in *Zinnia elegans*. J. Exp. Bot. 56, 2085–2093 10.1093/jxb/eri207 15955789

[B59] Kelliher T. Walbot V. (2012). Hypoxia triggers meiotic fate acquisition in maize. Science 337, 345–348 10.1126/science.1220080 22822150 PMC4101383

[B60] Kirchsteiger K. Ferrández J. Pascual M. B. González M. Cejudo F. J. (2012). NADPH thioredoxin reductase C is localized in plastids of photosynthetic and nonphotosynthetic tissues and is involved in lateral root formation in *Arabidopsis*. Plant Cell 24, 534–548 10.1105/tpc.111.092304 22505729 PMC3398562

[B61] Kirchsteiger K. Pulido P. González M. Cejudo F. J. (2009). NADPH Thioredoxin reductase C controls the redox status of chloroplast 2-Cys peroxiredoxins in *Arabidopsis thaliana*. Mol. Plan. 2, 298–307 10.1093/mp/ssn082 19825615

[B62] Klaas M. Yang B. Bosch M. Thorogood D. Manzanares C. Armstead I. P. (2011). Progress towards elucidating the mechanisms of self-incompatibility in the grasses: further insights from studies in Lolium. Ann. Bot. 108, 677–685 10.1093/aob/mcr186 21798860 PMC3170160

[B63a] Konrad K. R. Wudick M. M. Feijó J. A. (2011). Calcium regulation of tip growth: new genes for old mechanisms. Curr. Op. Plant Biol. 14, 721–730 10.1016/j.pbi.2011.09.005 22000040

[B63] Laloi C. Rayapuram N. Chartier Y. Grienenberger J. M. Bonnard G. Meyer Y. (2001). Identification and characterization of a mitochondrial thioredoxin system in plants. Proc. Natl. Acad. Sci. U.S.A. 98, 14144–14149 10.1073/pnas.241340898 11717467 PMC61182

[B64] Lee J. Y. Lee D. H. (2003). Use of serial analysis of gene expression technology to reveal changes in gene expression in *Arabidopsis* pollen undergoing cold stress. Plant Physiol. 132, 517–529 10.1104/pp.103.020511 12805584 PMC166994

[B65] Li X. M. Paech N. Nield J. Hayman D. Langridge P. (1997). Self-incompatibility in the grasses: evolutionary relationship of the S gene from *Phalaris coerulescens* to homologous sequences in other grasses. Plant Mol. Biol. 34, 223–232 10.1023/A:1005802327900 9207838

[B66] Li X. Nield J. Hayman D. Langridge P. (1994). Cloning a putative self-incompatibility gene from the pollen of the grass *Phalaris coerulescens*. Plant Cell 6, 1923–1932 10.2307/3869918 7866033 PMC160572

[B67] Li X. Nield J. Hayman D. Langridge P. (1995). Thioredoxin activity in the C terminus of Phalaris S protein. Plant J. 8, 133–138 10.1046/j.1365-313X.1995.08010133.x 7655504

[B68] Li X. Nield J. Hayman D. Langridge P. (1996). A self-fertile mutant of Phalaris produces an S protein with reduced thioredoxin activity. Plant J. 10, 505–513 10.1046/j.1365-313X.1996.10030505.x 8811864

[B69] Lind J. L. Bönig I. Clarke A. E. Anderson M. A. (1996). A style-specific 120-kDa glycoprotein enters pollen tubes of Nicotiana alata *in vivo*. Sex. Plant Reprod. 9, 75–86 10.1007/BF02153054

[B70] Lindermayr C. Saalbach G. Durner J. (2005). Proteomic indentification of S-nitrosylated proteins in *Arabidopsis*. Plant Physiol. 2005, 137, 921–930 10.1104/pp.104.058719 15734904 PMC1065393

[B71] Lindermayr C. Sell S. Muller B. Leister D. Durner J. (2010). Redox regulation of the NPR1-TGA1 system of *Arabidopsis thaliana* by nitric oxide. Plant Cell 22, 2894–2907 10.1105/tpc.109.066464 20716698 PMC2947166

[B72] Marri L. Zaffagnini M. Collin V. Issakidis-Bourguet E. Lemaire S. D. Pupillo P. (2009). Prompt and easy activation by specific thioredoxins of Calvin cycle enzymes of *Arabidopsis thaliana* associated in the GAPDH/CP12/PRK supramolecular complex. Mol. Plant. 2, 259–269 10.1093/mp/ssn061 19825612

[B73] Martinez A. Traverso J. A. Valot B. Ferro M. Espagne C. Ephritikhine G. (2008). Extent of N-terminal modifications in cytosolic proteins from eukaryotes. Proteomics 8, 2809–2831 10.1002/pmic.200701191 18655050

[B74] Márton M. L. Dresselhaus T. (2008). A comparison of early molecular fertilization mechanisms in animals and flowering plants. Sex. Plant Reprod. 21, 37–52 10.1007/s00497-007-0062-8

[B75] Marty L. Siala W. Schwarzländer M. Fricker M. D. Wirtz M. Sweetlove L. J. (2009). The NADPH-dependent thioredoxin system constitutes a functional backup for cytosolic glutathione reductase in *Arabidopsis*. Proc. Natl. Acad. Sci. U.S.A. 106, 9109–9114 10.1073/pnas.0900206106 19451637 PMC2690020

[B76] Mazzurco M. Sulaman W. Elina H. Cock J. M. Goring D. R. (2001). Further analysis of the interactions between the Brassica S receptor kinase and three interacting proteins (ARC1, THL1 and THL2) in the yeast two-hybrid system. Plant Mol. Biol. 45, 365–376 10.1023/A:1006412329934 11292081

[B77] McClure B. A. Franklin-Tong V. (2006). Gametophytic self-incompatibility: understanding the cellular mechanisms involved in “self” pollen tube inhibition. Planta 224, 233–245 10.1007/s00425-006-0284-2 16794841

[B78] McInnis S. M. Costa L. M. Gutiérrez-Marcos J. F. Henderson C. A. Hiscock S. J. (2005). Isolation and characterization of a polymer phic stigma-specific class III peroxidase gene from *Senecio squalidus* L. (Asteraceae). Plant. Mol. Biol. 57, 659–677 10.1007/s11103-005-1426-9 15988562

[B79] McInnis S. M. Desikan R. Hancock J. T. Hiscock S. J. (2006a). Production of reactive oxygen species and reactive nitrogen species by angiosperm stigmas and pollen: potential signalling crosstalk? New Phytol. 172, 221–228 10.1111/j.1469-8137.2006.01875.x 16995910

[B80] McInnis S. M. Emery D. C. Porter R. Desikan R. Hancock J. T. Hiscock S. J. (2006b). The role of stigma peroxidases in flowering plants: insights from further characterization of a stigma-specific peroxidase (SSP) from *Senecio squalidus* (Asteraceae). J. Exp. Bot. 57, 1835–1846 10.1093/jxb/erj182 16698818

[B81] Meng L. Wong J. H. Feldman L. J. Lemaux P. G. Buchanan B. B. (2010). A membrane-associated thioredoxin required for plant growth moves from cell to cell, suggestive of a role in intercellularcommunication. Proc. Natl. Acad. Sci. U.S.A. 107, 3900–3905 10.1073/pnas.0913759107 20133584 PMC2840455

[B82] Meyer Y. Belin C. Delorme-Hinoux V. Reichheld J. P. Riondet C. (2012). Thioredoxin and glutaredoxin systems in plants: molecular mechanisms, crosstalks, and functional significance. Antioxid. Redox. Signal. 17, 1124–1160 10.1089/ars.2011.4327 22531002

[B83] Miranda-Vizuete A. Sadek C. M. Jiménez A. Krause W. J. Sutovsky P. Oko R. (2004). The mammalian testis-specific thioredoxin system. Antioxid. Redox Signal. 6, 25–40 10.1089/152308604771978327 14713334

[B84] Murmu J. Bush M. J. DeLong C. Li S. Xu M. Khan M. (2010). *Arabidopsis* basic leuine-zipper transcription factors TGA9 and TGA10 interact with floral glutaredoxins ROXY1 and ROXY2 and are redundantly required for anther development. Plant Physiol. 154, 1492–1504 10.1104/pp.110.159111 20805327 PMC2971623

[B85] Ok S. H. Yoo K. S. Shin J. S. (2012). CBSXs are sensor relay proteins sensing adenosine-containing ligands in *Arabidopsis*. Plant Signal. Behav. 7, 664–667 10.4161/psb.19945 22580706 PMC3442862

[B86] Pahr S. Constantin C. Mari A. Scheiblhofer S. Thalhamer J. Ebner C. (2012). Molecular characterization of wheat allergens specifically recognized by patients suffering fromwheat-induced respiratory allergy. Clin. Exp. Allergy 42, 597–609 10.1111/j.1365-2222.2012.03961.x 22417217

[B87] Potocký M. Jones M. A. Bezvoda R. Smirnoff N. Ždárskı V. (2007). Reactive oxygen species produced by NADPH oxidase are involved in pollen tube growth. New Phytol. 174, 742–751 10.1111/j.1469-8137.2007.02042.x 17504458

[B88] Potocký M. Pejchar P. Gutkowska M. Jiménez-Quesada M. J. Potocká A. Alché J. D. (2012). NADPH oxidase activity in pollen tubes is affected by calcium ions, signaling phospholipids and Rac/Rop GTPases. J. Plant Physiol. 169, 1654–1663 10.1016/j.jplph.2012.05.014 22762791

[B89] Prado A. M. Colaço R. Moreno N. Silva A. C. Feijó J. A. (2008). Targeting of Pollen Tubes to Ovules Is Dependent on Nitric Oxide (NO) Signaling. Mol. Plant 1, 703–714 10.1093/mp/ssn034 19825574

[B90] Prado A. M. Porterfield D. M. Feijó J. A. (2004). Nitric oxide is involved in growth regulation and re-orientation of pollen tubes. Development 131, 2707–2714 10.1242/dev.01153 15128654

[B91] Pulido P. Spínola M. C. Kirchsteiger K. Guinea M. Pascual M. B. Sahrawy M. (2010). Functional analysis of the pathways for 2-Cys peroxiredoxin reduction in *Arabidopsis thaliana* chloroplasts. J. Exp. Bot. 61, 4043–4054 10.1093/jxb/erq218 20616155 PMC2935875

[B92] Reichheld J. P. Mestres-Ortega D. Laloi C. Meyer Y. (2002). The multigenic family of thioredoxin h in *Arabidopsis thaliana*: specific expression and stress response. Plant Physiol. Biochem. 40, 685–690 10.1016/S0981-9428(02)01406-7

[B93] Reichheld J. P. Khafif M. Riondet C. Droux M. Bonnard G. Meyer Y. (2007). Inactivation of thioredoxin reductases reveals a complex interplay between thioredoxin and glutathione pathways in *Arabidopsis* development. Plant Cell 19, 1851–1865 10.1105/tpc.107.050849 17586656 PMC1955716

[B94] Schopfer C. R. Nasrallah M. E. Nasrallah J. B. (1999). The male determinant of self-incompatibility in Brassica. Science 286, 1697–1700 10.1126/science.286.5445.1697 10576728

[B95] Sharma B. Bhatla S. C. (2013). Accumulation and scavenging of reactive oxygen species and nitric oxide correlate with stigma maturation and pollen–stigma interaction in sunflower. Acta Physiol. Plant. 35, 2777–2787 10.1007/s11738-013-1310-1

[B96] Shürmann P. Jacquot J. P. (2000). Plant thioredoxin systems revisited. Annu. Rev. Plant Physiol. Plant Mol. Biol. 51, 371–400 10.1146/annurev.arplant.51.1.371 15012197

[B97] Singh P. Kaloudas D. Raines C. A. (2008). Expression analysis of the *Arabidopsis* CP12 gene family suggests novel roles for these proteins in roots and floral tissues. J. Exp. Bot. 59, 3975–3398 10.1093/jxb/ern236 18974062 PMC2576635

[B98] Šírová J. Sedláøová M. Piterková J. Luhová L. Petøivalskı M. (2011). The role of nitric oxide in the germination of plant seeds and pollen. Plant Sci. 181, 560–572 10.1016/j.plantsci.2011.03.014 21893253

[B99] Spínola M. C. Pérez-Ruiz J. M. Pulido P. Kirchsteiger K. Guinea M. González M. (2008). NTRC new ways of using NADPH in the chloroplast. Physiol. Plant. 133, 516–524 10.1111/j.1399-3054.2008.01088.x 18346073

[B100] Sprunck S. Rademacher S. Vogler F. Gheyselinck J. Grossniklaus U. Dresselhaus T. (2013). Egg cell–secreted EC1 triggers sperm cell activation during double fertilization. Science 338, 1093–1097 10.1126/science.1223944 23180860

[B101] Tada Y. Spoel S. H. Pajerowska-Mukhtar K. Mou Z. Song J. Wang C. (2008). Plant immunity requires conformational changes of NPR1 via S-nitrosylation and thioredoxins. Science 321, 952–956 (Erratum in *Science* 325:1072). 10.1126/science.1156970 18635760 PMC3833675

[B102] Takasaki T. Hatakeyama K. Suzuki G. Watanabe M. Isogai A. Hinata K. (2000). The S receptor kinase determines self-incompatibility in Brassica stigma. Nature 403, 913–916 10.1038/35002628 10706292

[B103] Takayama S. Isogai A. (2005). Self-incompatibility in plants. Annu. Rev. Plant. Biol. 56, 467–489 10.1146/annurev.arplant.56.032604.144249 15862104

[B104] Taylor L. P. Hepler P. K. (1997). Pollen germination and tube growth. Annu. Rev. Plant Physiol. Plant Mol. Biol. 48, 461–491 10.1146/annurev.arplant.48.1.461 15012271

[B105] Timofejeva L. Skibbe D. S. Lee S. Golubovskaya I. Wang R. Harper L. (2013). Cytological characterization and allelism testing of anther developmental mutants identified in a screen of maize male sterile lines. G3 (Bethesda). 3, 231–249 10.1534/g3.112.004465 23390600 PMC3564984

[B106] Toriyama K. Hanaoka K. Okada T. Watanabe M. (1998). Molecular cloning of a cDNA encoding a pollen extracellular protein as a potential source of a pollen allergen in Brassica rapa. Febs Lett. 424, 234–238 10.1016/S0014-5793(98)00174-4 9539157

[B107] Traverso J. A. Meinnel T. Giglione C. (2008). Expanded impact of protein N-myristoylation in plants. Plant Signal. Behav. 3, 501–502 10.4161/psb.3.7.6039 19704499 PMC2634443

[B108] Traverso J. A. Micalella C. Martinez A. Brown S. C. Satiat-Jeunemaître B. Meinnel T. (2013). Roles of N-Terminal fatty acid acylations in membrane compartment partitioning: *Arabidopsis h*-Type thioredoxins as a case study. Plant Cell 25, 1056–1077 10.1105/tpc.112.106849 23543785 PMC3634677

[B109] Traverso J. A. Vignols F. Cazalis R. Pulido A. Sahrawy M. Cejudo F. J. (2007). PsTRX*h*1 and PsTRX*h*2 are both pea *h*-type thioredoxins with antagonistic behavior in redox imbalances. Plant Physiol. 143, 300–311 10.1104/pp.106.089524 17098852 PMC1761970

[B110] Utsumi T. Ohta H. Kayano Y. Sakurai N. Ozoe Y. (2005). The N-terminus of B96Bom, a *Bombyx mori* G-protein-coupled receptor, is N-myristoylated and translocated across the membrane. FEBS J. 272, 472–481 10.1111/j.1742-4658.2004.04487.x 15654885

[B111] Villarreal F. Martín V. Colaneri A. González-Schain N. Perales M. Martín M. (2009). Ectopic expression of mitochondrial gamma carbonic anhydrase 2 causes male sterility by anther indehiscence. Plant Mol. Biol. 70, 471–485 10.1007/s11103-009-9484-z 19326245

[B112] Wan C. Li S. Wen L. Kong J. Wang K. Zhu Y. (2007). Damage of oxidative stress on mitochondria during microspores development in Honglian CMS line of rice. Plant Cell Rep. 26, 373–382 10.1007/s00299-006-0234-2 17053903

[B113] Wang C. L. Wu J. Xu G. H. Gao Y. B. Chen G. Wu J. Y. (2010a). S-RNase disrupts tip-localized reactive oxygen species and induces nuclear DNA degradation in incompatible pollen tubes of *Pyrus pyrifolia*. J. Cell Sci. 123, 4301–4309 10.1242/jcs.075077 21098637

[B114] Wang S. Xie B. Yin L. Duan L. Li Z. Egrinya A. (2010b). Increased UV-B radiation affects the viability, reactive oxygen species accumulation and antioxidant enzyme activities in maize (*Zea mays* L.) Pollen. Photochem. Photobiol. 86, 110–116 10.1111/j.1751-1097.2009.00635.x 19906093

[B115] Wang Y. Chen T. Zhang C. Hao H. Liu P. Zheng M. (2009a). Nitric oxide modulates the influx of extracellular Ca2+ and actin filament organization during cell wall construction in *Pinus bungeana* pollen tubes. New Phytol. 182, 851–862 10.1111/j.1469-8137.2009.02820.x 19646068

[B116] Wang Z. Xing S. Birkenbihl R. P. Zachgo S. (2009b). Conserved functions of *Arabidopsis* and rice CC-type glutaredoxins in flower development and pathogen response. Mol. Plant 2, 323–335 10.1093/mp/ssn078 19825617

[B117] Wilkins K. A. Bancroft J. Bosch M. Ings J. Smirnoff N. Franklin-Tong V. E. (2011). Reactive oxygen species and nitric oxide mediate actin reorganization and programmed cell death in the self-incompatibility response of papaver. Plant Physiol. 156, 404–416 10.1104/pp.110.167510 21386034 PMC3091060

[B118] Wilson I. D. Hiscock S. J. James P. E. Hancock J. T. (2009). Nitric oxide and nitrite are likely mediators of pollen interactions. Plant Signal. Behav. 4, 416–418 10.4161/psb.4.5.8270 19816116 PMC2676752

[B119] Wu H. de Graaf B. Mariani C. Cheung A. Y. (2001). Hydroxyproline-rich glycoproteins in plant reproductive tissues: structure, functions and regulation. Cell Mol. Life Sci. 58, 1418–1429 10.1007/PL00000785 11693523 PMC11337276

[B120] Xing S. Zachgo S. (2008). ROXY1 and ROXY2, two *Arabidopsis* glutaredoxin genes, are required for anther development. Plant J. 53, 790–801 10.1111/j.1365-313X.2007.03375.x 18036205

[B121] Xing S. Lauri A. Zachgo S. (2006). Redox regulation and flower development: a novel function for glutaredoxins. Plant Biol. 8, 547–555 10.1055/s-2006-924278 16883479

[B122] Xing S. Rosso M. G. Zachgo S. (2005). ROXY1, a member of the plant glutaredoxin family, is required for petal development in *Arabidopsis thaliana*. Development 132, 1555–1565 10.1242/dev.01725 15728668

[B123] Xing S. Salinas M. Huijser P. (2011). New players unveiled in early anther development. Plant Signal. Behav. 6, 934–938 10.4161/psb.6.7.15668 21633200 PMC3257765

[B124] Yoo K. S. Ok S. H. Jeong B. C. Jung K. W. Cui M. H. Hyoung S. (2011). Single cystathionine β-synthase domain-containing proteins modulate development by regulating the thioredoxin system in *Arabidopsis*. Plant Cell 23, 3577–3594 10.1105/tpc.111.089847 22021414 PMC3229136

[B125] Yun B. W. Feechan A. Yin M. Saidi N. B. Le Bihan T. Yu M. (2011). S-Nitrosylation of NADPH oxidase regulates cell death in plant immunity. Nature 478, 264–268 10.1038/nature10427 21964330

[B126] Zafra A. Rodríguez-García M. I. Alché J. D. (2010). Cellular localization of ROS and NO in olive reproductive tissues during flower development. BMC Plant Biol. 10:36 10.1186/1471-2229-10-36 20181244 PMC2838403

[B127] Zaidi M. A. O'Leary S. Wu S. Gleddie S. Eudes F. Laroche A. (2012). A molecular and proteomic investigation of proteins rapidly released from triticale pollen upon hydration. Plant Mol. Biol. 79, 101–121 10.1007/s11103-012-9897-y 22367549

[B128] Zechmann B. Koffler B. E. Russell S. D. (2011). Glutathione synthesis is essential for pollen germination *in vitro*. BMC Plant Biol. 11:54 10.1186/1471-2229-11-54 21439079 PMC3078877

